# Electrochemical biosensors: a novel approach for rapid and cost-effective hCG monitoring in gestational trophoblastic tumours

**DOI:** 10.1039/d5ra09483f

**Published:** 2026-05-13

**Authors:** Yuspian Nur, Harra Ismi Farah, Wahyu Widayat, Andi Kurniadi, Gatot Nyarumenteng Adipurnawan Winarno, Yudi Mulyana Hidayat, Kemala Isnainiasih Mantilidewi, Yeni Wahyuni Hartati, Toto Subroto, Muhammad Yusuf

**Affiliations:** a Research Center for Molecular Biotechnology and Bioinformatics, Universitas Padjadjaran Bandung 40132 West Java Indonesia; b Research Centre for Electronics, National Research and Innovation Agency (BRIN) KST Samaun Samadikun Bandung 40135 West Java Indonesia; c Department of Pharmaceutical Sciences, Faculty of Pharmacy, Universitas Mulawarman Samarinda 75242 East Kalimantan Indonesia; d Department of Obstetrics and Gynecology, Universitas Padjadjaran Bandung 40161 West Java Indonesia; e Department of Chemistry, Universitas Padjadjaran Bandung 45363 West Java Indonesia m.yusuf@unpad.ac.id

## Abstract

Human chorionic gonadotropin (β-hCG) is a key biomarker for the diagnosis and monitoring of gestational trophoblastic disease (GTD). Current gold standard methods, such as chemiluminescent immunoassays and ELISA, are limited by high operational costs, long analysis times, and the requirements for centralized laboratory facilities, which restrict their accessibility, especially in resource-limited settings. As a promising alternative, electrochemical biosensors offer rapid, sensitive, selective, and cost-effective β-hCG detection, suitable for point-of-care applications. This review summarizes recent advances in the development of electrochemical β-hCG biosensors, with an emphasis on innovations in electrode engineering, interface design, biological recognition elements, and signal amplification strategies that significantly improve analytical performance, including detection limits and linear dynamic range. Considerable focus is assigned to biosensors that are capable of accurately measuring β-hCG at high concentrations, as required for effective clinical management of GTD. The review also discusses major challenges, such as prolonged stability, selectivity in complex biological matrices, reproducibility, and consistent measurement performance at clinically relevant high β-hCG levels. Finally, emerging trends such as miniaturization, microfluidic integration, and multiple detection are highlighted as promising directions to support real-time GTD monitoring.

## Abstract 2

Electrochemical β-hCG biosensors are reviewed with emphasis on signal amplification strategies that extend linear detection ranges, enabling improved detection of high biomarker levels for gestational trophoblastic disease monitoring.

## Graphical abstract

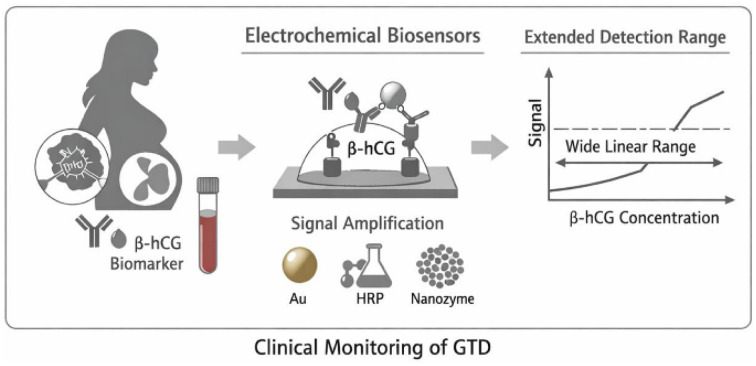

## 1 Introduction

Point-of-care (PoC) diagnostics have garnered significant attention in the medical field due to their ability to enable real-time, remote, and accurate health monitoring. These systems facilitate the rapid detection of diseases directly at or near the patient's location, thereby reducing reliance on both traditional and modern laboratory-based diagnostics. By providing immediate test results, PoC devices support faster clinical decision-making, which can lead to timely interventions and improved patient outcomes. The simplicity, portability, and cost-effectiveness of PoC technology make it particularly valuable in resource-limited settings, where access to advanced diagnostic facilities is often restricted.^1,2^ Additionally, ongoing advancements in PoC technology—such as improvements in sensitivity, specificity, and integration with digital healthcare platforms—are further enhancing its role in modern medicine. As these technologies continue to evolve, they hold immense potential to revolutionize disease detection and management by offering rapid, reliable, and widely accessible diagnostic solutions.^3^

As point-of-care (PoC) technology continues to evolve, the demand for improved diagnostic solution in gynaecologic oncology has increased. GTD, particularly gestational trophoblastic neoplasia (GTN), represent a clinical condition in which early detection and continuous monitoring are essential for effective disease management. Epidemiological evidence indicate that the prevalence of GTD is substantially higher in Asian populations, occurring in approximately 1 in 400 pregnancies, compared with 1 in 1500 to 2000 pregnancies in Europe and the United States.^4,5^ Moreover, certain population demonstrate increased susceptibility to more aggressive disease phenotypes.^6,7^ Despite its relative rarity on a global scale, GTN poses a significant threat to maternal health, particularly in resource-limited setting.^8–10^ In addition to documented geographic and ethnics disparities, GTD remains associated with a substantial clinical burden. Mortality rate has reached 4.8% in certain cohorts, largely attributed to delayed diagnosis and restricted access to specialized care, which contribute to advance disease staging and poorer outcomes.^11^ Similarly, choriocarcinoma mortality rates approaching 10% have also been documented despite the availability of chemotherapy.^12^

GTD encompasses a spectrum of trophoblastic proliferative disorders ranging from benign hydatidiform mole to malignant GTN.^13–15^ Low-risk GTN typically responds favourably to single-agent chemotherapy, with remission rates approaching 77% and excellent long-term survival.^16^ In contrast, more aggressive variants exhibited comparatively lower survival rates, with reported 5- and 10-year survival of approximately 80% and 75%, respectively.^17^

The primary treatment for GTN is chemotherapy, with surgical intervention reserved for resistance cases to achieve complete disease eradication.^17^ Cisplatin-based and high-dose chemotherapy regimens have demonstrated the improvement of survival outcomes in patients with poor prognostic factors. Clinical outcomes are influences by several variables, including an interval of more than 48 months since the antecedent pregnancy and a WHO risk score of 5–6, both of which are associated with increased treatment resistance and the need for more intensive therapy.^16,18^ In refractory case or when severe complication such as uncontrollable bleeding occur, hysterectomy may be considered, especially in patient who no longer desire fertility.^17^

Despite its relatively low incidence, GTN may progress rapidly and exhibited aggressive behaviour if not detected at an early stage, delayed diagnosis lead to more severe disease stage, increasing complication such as haemorrhage and uterine perforation, as well as metastases most commonly to the lungs and brain.^19^ Such progression, is strongly associated with poorer prognosis, heightened maternal morbidity and increased risk of mortality.^20,21^ These clinical implications emphasize the importance of sensitive and reliable diagnostic platform that facilitate early detection and continuous monitoring. This objective depends on the accurate quantification of molecular biomarkers reflecting trophoblastic activity.

Human chorionic gonadotropin (hCG) is a heterodimeric glycoprotein hormone composed of non-covalently associated α- and β-subunit, where the α-subunit is common to other glycoprotein hormones (LH, FSH, and TSH), and the β-subunit confers biological and immunological specificity. In normal pregnancy, intact hCG (α + β) is the predominant circulating form responsible for maintaining corpus luteum function.^22^ By contrast, β-hCG refers to the free β-subunit that circulates independently and is typically present at much lower concentration under physiological condition.^23^ However, disproportionate secretion of free β-hCG may occur in trophoblastic disorders and certain germ cell tumours. Owing to this pathological overexpression, β-hCG serves as the primary biomarker for diagnosing and monitoring GTT. Elevated β-hCG serum levels are often the first indication of this condition, with concentrations ranging from 1000 to over 100 000 mIU mL^−1^, depending on the type and stage of the disease.^24,25^ Accurate β-hCG detection is essential, as early diagnosis facilitates prompt medical intervention, which directly improves patient prognosis and survival rates.^26^ In clinical practice, serial β-hCG measurements are routinely performed to assess disease progression, evaluate treatment efficacy, and detect potential relapse following therapy.

Several laboratory-based techniques have been developed for β-hCG detection, with chemiluminescent immunoassays (CLIA) and enzyme-linked immunosorbent assays (ELISA) being the most widely used methods. CLIA provides high sensitivity through chemiluminescence-based detection, while ELISA relies on antigen–antibody interactions to quantify β-hCG in blood or urine samples. These methods are highly effective for early diagnosis and disease monitoring; however, they have inherent limitations, including high operational costs, lengthy processing times, and the requirement for specialized laboratory infrastructure.^24^

Despite their status as gold-standard techniques, conventional methods such as CLIA and ELISA have several drawbacks, particularly in resource-limited settings. These techniques can be expensive and time-consuming, often requiring complex sample preparation, invasive sampling procedures, and extended processing times. Moreover, these methods demand well-equipped laboratories and trained personnel, limiting their accessibility in low-resource areas.^27–29^ Such constraints delay timely diagnosis and complicate follow-up assessments, which are crucial for effective GTN management. Given the growing demand for functional and accessible medical diagnostics, there is an urgent need to explore alternative diagnostic strategies that are more efficient, cost-effective, and widely available.

In response to these challenges, alternative diagnostic systems that enable rapid, sensitive, and cost-effective β-hCG detection have gained increasing attention. Electrochemical biosensors have emerged as a promising low-cost, rapid, and accurate alternative for β-hCG detection, offering a minimally invasive and user-friendly approach suitable for point-of-care (PoC) applications.^30^ These biosensors provide high sensitivity, selectivity, and rapid detection, while minimizing the need for extensive sample preparation, thereby addressing key challenges faced by both patients and healthcare providers. The integration of electrochemical biosensors into clinical laboratories holds substantial potential for enhancing GTN diagnosis and treatment monitoring.^24,31,32^ This technology has the potential to complement or even replace traditional methods in specific clinical scenarios, particularly for real-time patient monitoring and use in resource-limited settings. However, current biosensor platforms still face technical limitations, particularly in achieving the required detection threshold for monitoring GTN, which necessitates β-hCG quantification at concentrations exceeding 50 000 mIU mL^−1^.

However, while current electrochemical biosensor offers rapid and sensitive β-hCG detection, most are optimized for pregnancy-level concentration and do not reliably cover the very high levels (>500 000–100 000 mIU mL^−1^) required for GTN monitoring. This critical gap motivates a systematic evaluation of reported biosensor platforms in the context of clinically relevant hCG ranges. Beyond conventional sensitivity-focused comparisons, this review maps electrode materials, nanomaterial architecture, biorecognition elements, and signal amplification strategies to their ability to expand linear dynamic range and maintain quantitative performance in complex biological matrices. By establishing a clinically driven, mechanistically integrated framework, it links sensor design directly to diagnostic decision thresholds, highlight strategies to mitigate signal saturation at elevated antigen levels, and offers a clear roadmap for the development of next-generation, rapid cost effective electrochemical β-hCG biosensors tailored for GTN management.

Furthermore, insight from this review can guide the development of portable, user-friendly electrochemical β-hCG biosensor, enhancing diagnostic accessibility in resource-limited setting. As biosensing technologies continue to advance, future platforms are expected to become faster, more precise, and more accessible to diverse populations, ultimately strengthening global healthcare systems.

## 2 Overview of gestational trophoblastic disease (GTD)

**Fig. 1:**
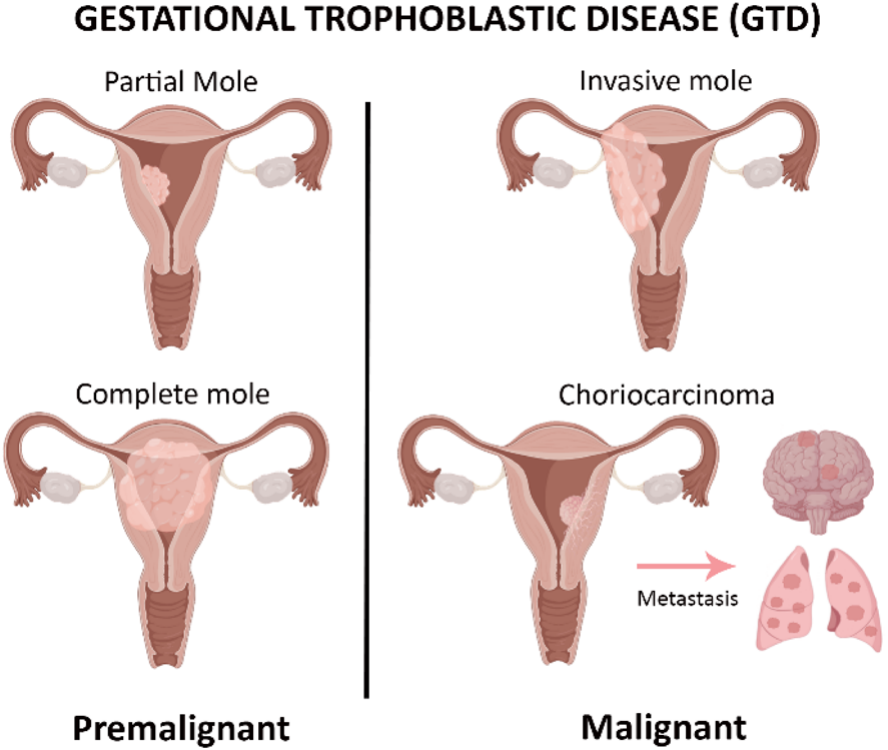
Classification of GTD into premalignant and malignant forms, representing abnormal trophoblastic cell growths in the placenta.

GTD comprises a heterogeneous group of rare disorders arising from abnormal proliferation of placental trophoblastic cells, which are responsible for embryo implantation and placental development.^33^ These conditions encompass a broad clinical spectrum ranging from premalignant lesions, such as complete and partial hydatidiform moles to malignant entities collectively referred to GTN. GTN includes invasive mole, choriocarcinoma, PSTT, and ETT (Fig. 1).^24,33^ These tumours exhibited diverse biological characteristics, varying from lesion that may regress spontaneously to highly aggressive malignancies with metastatic potential.

An invasive mole is defined by the penetration of molar villi into the uterine myometrium, whereas choriocarcinoma is a highly malignant tumour with a strong propensity for early hematogenous metastasis, most frequently involving the lungs and brain. PSTT and ETT are relatively rare subtypes originating from intermediate trophoblastic cells and demonstrate distinct clinical and pathological features.

The global incidence of GTD varies across geographic regions, generally ranging from approximately 0.6 to 1.1 cases per 1000 pregnancies.^34^ Epidemiological studies indicate a higher prevalence in Asians population compared with Western countries. Molar pregnancy, the primary precursor to GTD, occurs in approximately 1 in 600 pregnancies worldwide, although significantly higher rates have been reported in certain regions of Asia.^35–37^ These geographic differences are believed to be influenced by a combination of genetic, environmental, and socioeconomic factors, as well as disparities in healthcare access.

Several risk factors have been associated with the development of GTD. Maternal age is one of the most significant determinants, with women older than 35 years particularly those over 40 showing a markedly increased risk compared with younger women. Conversely, pregnancies occurring before the age of 20 have also been associated with elevated risk, suggesting that hormonal and reproductive factors both extremes of reproductive age may contribute to disease pathogenesis.^6,24,38^ A previous history of hydatidiform mole represents another important risk factor, with recurrence rate estimated at approximately 1%, which is substantially higher than that observed in the general population. Additional factors, including genetic predisposition, multiparity, nutritional deficiencies, and environmental exposures, have also been suggested to contribute to disease development, although the precise mechanism remain under investigation.^10,39,40^

One of the major challenges in the management of GTD is the variability in their clinical presentation, including PSTT and ETT.^17,41^ Each of these conditions has distinct histopathological characteristics and clinical behaviours, necessitating a comprehensive understanding of their features for effective diagnosis and treatment.^42^ For instance, PSTT is extremely rare, accounting for only 0.2–2% of gestational trophoblastic disease cases, and can arise following various gestational events, including normal pregnancy and miscarriage.^43^ Understanding the epidemiology and associated risk factors is crucial for early detection and optimal management of GTD, ultimately reducing morbidity and improving patient prognosis.

The monitoring of GTT often involves measuring serum human chorionic gonadotropin (hCG) levels, which serves as a tumour marker for these conditions. Elevated hCG levels may indicate the presence of GTN, and serial monitoring of hCG levels helps assess treatment response and detect recurrence.^44,45^ However, interpreting hCG levels can be complex due to factors such as the timing of measurement and the presence of other conditions affecting hCG production.^25^ Therefore, the development of standardized protocols for hCG monitoring is crucial to improving diagnostic accuracy and patient outcomes.

In addition to hCG monitoring, imaging studies such as ultrasonography, computed tomography (CT), and magnetic resonance imaging (MRI) play an important role in evaluation of GTT. These techniques facilitate the assessment of disease extent, detection of metastatic lesions, and support clinical decision-making for appropriate treatment strategies.^46^ However, access to advanced imaging technologies remains limited in many healthcare setting, particularly in resourced-constrained regions, highlighting the need for cost effective and reliable diagnostic alternatives.

Management of GTN generally involves a combination of surgical intervention and chemotherapy. Surgical procedures may include evacuation of a molar tissue or tumour resection, whereas chemotherapy is the primary treatment for malignant forms such as choriocarcinoma. The multi-agent EMA/CO regimen is commonly used and has demonstrated high therapeutic efficacy, even in metastatic disease.^47,48^

In addition to clinical challenge, GTT may impose substantial physiological distress on patients. Concern regarding fertility and future reproductive outcomes are particularly prominent among younger women following diagnosis.^49,50^ Accordingly, increasing attention has been directed toward fertility-preserving therapeutic strategies that enable effective disease control while maintaining reproductive potential.^17,51^ These considerations emphasize the importance of multidisciplinary management that integrates both medical treatment and psychosocial support.

Accurate diagnosis and effective monitoring are essential for optimal management of GTD. Ultrasonography combined with measurement of serum hCG levels remains the primary approach for both diagnosis and post-treatment surveillance of GTD, play a critical role in early detection and ongoing patient management.^52^

Ultrasonographic imaging enables visualization of intrauterine contents and facilitate differentiation between complete and partial hydatidiform moles. Complete moles typically present with the characteristic “snowstorm” appearance caused by multiple cystic spaces, whereas partial moles may demonstrate the presence of fetal tissue accompanied by abnormal placental tissue. Additionally, intravaginal Doppler ultrasound can assist in lesion localization and assessment of malignant potential by evaluating local invasion, thereby contributing to disease staging.^53^

Elevated or persistently rising hCG levels may indicate the presence of persistent trophoblastic disease or the development of GTN. Serial monitoring of hCG concentration is therefore routinely performed to evaluate treatment response and detect disease recurrence at an early stage.^8,54^ Clinical guidelines (The National Comprehensive Cancer Network, NCNN) recommend regular hCG measurement following molar pathology, as approximately 15% of complete hydatidiform moles may progress to malignant trophoblastic neoplasia.^14,54^

Risk stratification is another critical component of GTN management. Patient are commonly classified according to the FIGO scoring system, which incorporates factors such as serum hCG levels, interval since antecedent pregnancy, tumour size, and presence of metastasis. Based on this scoring system, patients are categorized into low-risk and high-risk groups to guide treatment selection.

Treatment strategies for GTN are primarily based on chemotherapy and is guided by FIGO/WHO risk stratification. Patient with low-risk disease (FIGO/WHO score 0–6) are typically treated single-agent chemotherapy, most commonly methotrexate or actinomycin-D, achieving remission rates approaching 100%. However, approximately 25–30% of patients with a FIGO score 5–6 develop resistance to first-line therapy, necessitating a more intensive treatment strategy.^55–57^ Additional factors such as high pre-treatment hCG levels and the presence of metastases may further worsen prognosis. Consequently, serial monitoring of hCG levels is crucial to assess treatment response and detect early recurrence.^58^

Patients with the high-risk category (FIGO/WHO score 7–12) have a greater likelihood of resistance to single agent therapy. Consequently, this group requires a more complex therapeutic approach, such as multi-agent chemotherapy regimens, which improve remission rates and reduce replace risk.^59,60^ Nevertheless, treatment success remains lower and therapy duration is often longer a longer compared with low-risk patiens.^61^

The ultra-high-risk patients (FIGO/WHO score ≥13) represent the most challenging group, frequently exhibited resistance to standard therapy and a high risk of recurrence. Management typically requires intensive multi-agent chemotherapy, often combined with surgical intervention or additional therapeutic modalities. Emerging strategies such as immunotherapy are also being explored for patients with poor responses to conventional chemotherapy. The high rate of early mortality observed in this group, particularly in resource-limited settings, highlights the importance of early diagnosis and access to optimal treatment facilities.^59–61^

Despite the high curability of GTN when appropriately treated, delayed diagnosis and limited access to healthcare services remain significant challenges, particularly in resource-limited settings. Early detections and continuous monitoring are essential to prevent disease progression and improve patient outcomes. These clinical needs highlight the importance of reliable, sensitive, and accessible diagnostic tools capable of facilitating timely detection and longitudinal monitoring of trophoblastic disease.

## 3 Challenging current GTD diagnostic methods and the importance of hCG monitoring

The primary diagnosis and management approach of GTD relies on the measurement of serum β-hCG levels, which serve as the key biomarker for disease detection, therapeutic monitoring, and recurrence surveillance. β-hCG is physiologically produced by trophoblastic cells during normal pregnancy. However, abnormally elevated levels are strongly associated with trophoblastic pathologies, particularly GTN. Consequently, quantitative measurement of β-hCG levels plays a central role in both the diagnosis and monitoring of patients with trophoblastic tumours.^62,63^ β-hCG levels also serve as important biomarkers for distinguishing among different types of germ cell tumours, including germinomas and non-germinomatous germ cell tumours (NGCT).^64^ In Japan, defined β-hCG threshold values (200 and 50 mIU mL^−1^) are used to guide clinical decisions in diagnosis and management of this tumour.^65^ In addition, β-hCG measurement is crucial in identifying gestational conditions such as hydatidiform moles, where the preeclampsia may occur despite the absence of a fetus, highlighting the hormone's role in predicting and diagnosing pregnancy-related complication.^63^

Abnormal β-hCG levels can have significant implications for disease management and therapeutic monitoring. In ectopic pregnancy, serial assessment of β-hCG levels is widely used to evaluate the effectiveness of medical treatment, particularly methotrexate therapy. A progressive decline in β-hCG levels following treatment generally indicates successful resolution, whereas persistently elevated concentrations may suggest treatment failure and necessitate further intervention.^66,67^ Beyond pregnancy-related disorders, increased β-hCG levels are also associated with several malignancies, including choriocarcinoma, where the hormone function as a biomarker of tumour activity and assist in guiding treatment strategies.^62^ Moreover, the clinical significance of β-hCG extend to its prognostic utility in reproductive medicine, initial β-hCG concentration have been reported correlate with the success of *in vitro* fertilization (IVF), with defined threshold values correlating with clinical pregnancy rates.^68,69^ This findings highlight the broader role of β-hCG not only in diagnosis but also in predicting treatment outcomes and supporting patient counselling and clinical management.

Although the role of β-hCG in diagnosing and management of GTN is well-established, further research and clinical trials are necessary to optimize its application in clinical practice. In particular, investigating the dynamics of β-hCG levels in different clinical conditions, such as following embryo transfer or during treatment for ectopic pregnancy, can provide deeper insights into its prognostic capabilities.^70,71^ Furthermore, a deeper understanding of the biochemical pathways and mechanisms regulating β-hCG production in both normal and pathological state could contribute to the development of more effective diagnostic and therapeutic strategies.^72^

The most commonly used methods for detecting β-hCG are chemiluminescent immunoassay (CLIA) and enzyme-linked immunosorbent assay (ELISA). These techniques have high sensitivity and specificity in detecting β-hCG, making them highly reliable in clinical practice. These techniques utilize the interaction between specific antibodies and antigens, in this case, β-hCG, to generate a measurable signal that is then used to assess the presence or concentration of the hormone in the patient's serum.

From a clinical and analytical perspective, the decision-making stages based on hCG levels (particularly in the management of GTD) set different requirements for detection range, sensitivity, and quantitative precision. At the early diagnosis stage, including early pregnancy screening and initial clinical evaluation of GTD, high sensitivity and low detection limits are required to reliably measure hCG levels at low to medium concentrations (Table 1) and clearly distinguish pathological increases based on the measured levels.^73^ For therapeutic monitoring during chemotherapy or other treatment, detection methods with a wide linear dynamic range and high signal linearity are required because hCG/β-hCG levels are very high. At this stage, clinical decision-making relies on trend analysis and accurate quantification of high hCG levels to assess response to therapy.^74^ Furthermore, detecting recurrence and post-treatment monitoring require increased sensitivity and precision in measuring very low hCG/β-hCG levels, approaching baseline values or nearly undetectable levels. This is useful for early identification of residual or recurrent disease. Failure to accommodate these varying analytical demands (particularly the limitations of the dynamic range at high concentrations) can lead to signal saturation, misinterpretation of disease status, and delays in clinical intervention. Therefore, only a few methods can optimally meet all clinical objectives, necessitating a hCG detection approach specifically designed and evaluated within the clinical decision-making pathway, with the right balance between sensitivity, linear dynamic range, and quantitative precision.

**Table 1:** hCG levels in various conditions

Condition		hCG levels	Ref.
Early pregnancy (3–4 weeks)		22–239 mIU mL^−1^	73
General pregnancy (6 weeks – term)		16.850 mIU mL^−1^	73
Ectopic pregnancy	Patients with an ectopic mass or fluid in the pouch of Douglas	>1500 mIU mL^−1^	73
Ectopic pregnancy	Patients without an ectopic mass or fluid in the pouch of Douglas	>2000 mIU mL^−1^	73
Complete hydatidiform mole		>100.000 mIU mL^−1^	73
Partial hydatidiform mole		Median: 48.900 mIU mL^−1^ (range of 11.600 to 220.114 mIU mL^−1^)	73
Choriocarcinoma		>1.000.000 mIU mL^−1^	73
Placental site trophoblastic tumour		Median: 30 mIU mL^−1^ (range of 1 to 231 mIU mL^−1^)	73
GTD		50.000–100.000 mIU mL^−1^	74
GTD	With molar disease	Higher than 10 000 mIU mL^−1^ at 5 weeks, 1000 mIU mL^−1^ at 8 weeks, or detectable at 24 weeks	74
Germ cell tumours	Seminomas	Increased by approximately 15–20%	82
Germ cell tumours	Non-seminomatous germ cell tumours (NSCGT)	Increased by approximately 40–50%, >5000 mIU mL^−1^ (good risk), 5000–50 000 mIU mL^−1^ (intermediate risk) and >50 000 mIU mL^−1^ (poor risk)	82 and 83
Transitional cell carcinoma (TCC)	Bladder cancer	Increased by approximately 10–75%	84
Prostate cancer		200–30 000 mIU mL^−1^	85

The primary advantage of immunoassay-based methods such as CLIA and ELISA is their ability to detect β-hCG with high precision, even at very low concentrations. This is particularly important in the context of GTN, where early diagnosis is critical for successful treatment and prognosis. Several studies have showed that these techniques consistently provide accurate results in detecting β-hCG, thus establishing them as the gold standard in monitoring and managing patients with GTN.^75,76^

While CLIA and ELISA are highly reliable for detecting biomarkers and exhibit high sensitivity, they have inherent limitations that can significantly impact clinical diagnostics. These methods often require a relatively long time to process samples, taking hours or even days to yield results. Such delays can hinder timely diagnosis and treatment, especially in critical medical situations.^77^ Furthermore, the complexity of sample preparation for these tests often requires invasive procedures, such as venous blood draws, which can be inconvenient and uncomfortable for patients.^78^

In addition to the time and discomfort associated with traditional immunoassays, there are also financial implications. These tests can be costly due to the need for specialized equipment and highly trained personnel to perform the tests accurately.^79^ The reliance on complex protocols and the potential for human error during sample handling and analysis further complicate the clinical utility of these traditional methods.^80^ Additionally, the specificity of immunoassays can be a concern, as cross-reactivity with similar biomolecules may lead to false-positive or false-negative results, complicating the interpretation of findings.^81^

The limitations of traditional immunoassays emphasize the need for more efficient and user-friendly diagnostic tools. As a result, there is a growing demand for the development of faster, more affordable methods that maintain the same high level of sensitivity and specificity in detecting β-hCG. Advances in biosensor technology, particularly electrochemical biosensors, offer a promising alternative that can address these challenges. These newer technologies can provide rapid, sensitive, and specific biomarker detection with minimal sample preparation and lower costs, making them ideal for point-of-care applications.^86,87^

## 4 Principles and advantages of electrochemical biosensors

**Fig. 2:**
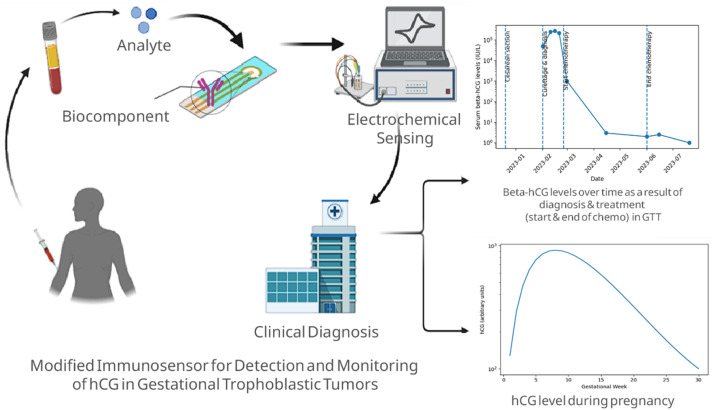
Schematic illustration of a clinical modified electrochemical immunosensor for β-hCG detection in GTD. β-hCG in blood samples binds to immobilized bioreceptor on a modified electrode, producing an electrochemical signal that converted into quantitative output, enabling dynamic monitoring of β-hCG levels during diagnosis and treatment.

Electrochemical biosensors have attracted significant attention in various fields, including environmental monitoring, food safety, and healthcare, due to their rapid analytical response, exceptional sensitivity, and potential for device miniaturization.^30^ In the field of clinical diagnostic, this platform is highly promising for the detection of hCG/β-hCG, an important biomarker for pregnancy screening, GTD/GTN, and monitoring therapy for these diseases. This device combines the immunological specificity of biological recognition elements, including antibodies or aptamers, with the sensitive transduction properties of an electrochemical system. Specifically, the selective interaction between β-hCG and its bioreceptor is converted into a measurable electrical signal, enabling quantitative analysis with high precision and reproducibility. The integration of selective antigen–bioreceptor recognition with electrochemical detection facilitates the reliable quantification of low-abundance biomarkers such as β-hCG. This supports the development of portable diagnostic platforms suitable for point-of-care testing and real-time monitoring.^88,89^ To better illustrate the potential clinical application of electrochemical biosensors for β-hCG monitoring, a conceptual workflow is presented in Fig. 2.

**Fig. 3:**
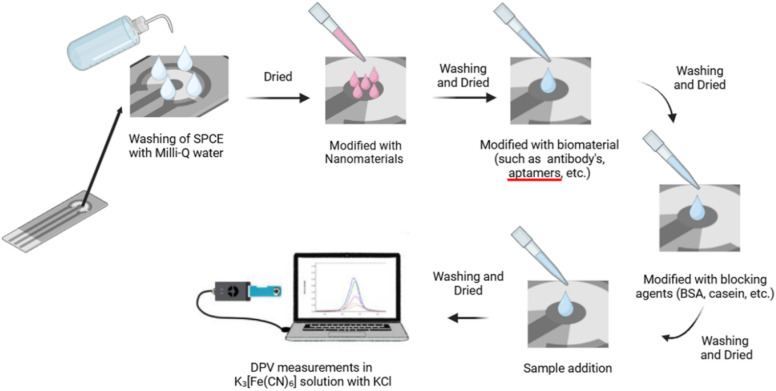
Electrode modification scheme with nanoparticles and biomaterials in electrochemical biosensors.

Measurement of hCG/β-hCG levels in a clinical context demands high accuracy and sensitivity, particularly because fluctuations in its concentration are often directly related to diagnostic determination, disease progression, and therapy effectiveness in patients with GTD and GTN. In its application, the serial monitoring of these biomarkers is necessary to evaluate the response to treatment and detect the possibility of disease persistence or recurrence. Therefore, analytical methods capable of providing quantitative results quickly, reliably, and repeatedly become crucial to support accurate clinical decision-making. Electrochemical biosensors are increasingly being explored due to their advantages over conventional immunological methods such as radioimmunoassay (RIA), ELISA, and chemiluminescent immunoassay (CLIA). Various biosensor designs that combine antibody capture systems, aptamer-based recognition, and nanostructured electrodes have been reported to detect hCG/β-hCG, as illustrated in Fig. 3.

Compared to traditional laboratory immunoassays, electrochemical biosensors offer a simplified analytical workflow and reduce operational complexity, typically requiring minimal sample preparation and simple instrumentation, as well as providing cost-efficient analysis.^90,91^ This operational simplicity facilitates broader implementation in primary healthcare services and decentralized healthcare service networks, thereby expanding access to essential diagnostic testing. In addition, this platform is designed for high analytical sensitivity and a wide linear detection range, allowing for accurate measurement of hCG/β-hCG at concentrations relevant to various clinical scenarios.^90,92,93^

In several reports, electrochemical biosensors show higher analytical sensitivity compared to conventional immunological methods, which is a crucial factor for detecting biomarkers at very low concentrations. This high sensitivity allows for the early identification of changes in biomarker levels that might be missed by standard methods. For example, several electrochemical biosensor platforms have been reported to detect β-hCG at concentrations as low as 0.00286 mIU mL^−1^, which is below the typical detection limit of the ELISA method. This capability is highly relevant in clinical contexts that require early monitoring and high precision, such as early pregnancy detection, evaluation of trophoblastic disease residue, and monitoring therapy response in the early stages, when β-hCG levels are still very low.^88,89^

However, the increase in high sensitivity needs to be balanced with adequate analytical specificity. One of the main challenges in detecting hCG/β-hCG lies in achieving high specificity in complex biological matrices. High specificity is necessary to ensures that the measured signal truly originates from hCG/β-hCG and not from interfering molecules in the biological samples.^92,94^ These limitations are often caused by cross-reactivity with hormones or other compounds that have similar structural or electrochemical properties. Electrochemical biosensors address this challenge by using carefully selected bioreceptors and optimizing the electrode surface chemistry to minimize interference from hormones with similar structures, such as luteinizing hormone (LH), follicle-stimulating hormone (FSH), and thyroid-stimulating hormone (TSH), [as well as non-specific interfering molecules] such as folic acid, ascorbic acid, bovine serum albumin (BSA), and l-glutamic acid. This strategy significantly reduces the likelihood of false positive results and improves diagnostic accuracy.^88,89,95,96^

Another advantage of electrochemical biosensors is their ability to offer a relatively wider linear detection range compared to the ELISA method. This wide linear range allows for the measurement of β-hCG at various concentration levels, from very low to very high, making it relevant for a range of clinical conditions. In applications such as pregnancy monitoring or the detection and follow-up of trophoblastic diseases, β-hCG concentrations can vary significantly over time. Therefore, the ability of electrochemical biosensors to maintain accuracy over a wide concentration range is crucial in supporting reliable clinical monitoring and precise medical decision-making.^88,97^ Nevertheless, the reported limits of the linear detection range in several studies of electrochemical biosensors for hCG/β-hCG still require improvement. As shown in Table 1, the performance of the linear range on several biosensor platforms does not yet fully encompass all relevant clinical concentration variations. These findings confirm that the expansion of the linear range, along with the overall improvement of analytical performance, is another major challenge in the development of electrochemical biosensors for the detection of hCG/β-hCG in clinical applications.

The analytical performance of electrochemical biosensors is greatly influenced by the selection of electrode materials and surface modification strategies. Gold and carbon-based electrodes are widely used due to their excellent electrical conductivity and biocompatibility. Surface engineering approaches, including the use of self-assembled monolayers (SAM) on gold electrodes, have been shown to enhance the efficiency of bioreceptor immobilization and signal stability. Research by Koterwa *et al.* (2023) and Dąbrowski *et al.* (2019) has shown that gold electrodes modified with SAM result in more sensitive and specific biosensors for β-hCG detection.^97,98^

Overall, the higher speed, sensitivity, detection range, and specificity make electrochemical biosensors a highly competitive and superior option for clinical applications. In many cases, especially those requiring early and accurate detection, such as in gestational trophoblastic disease or pregnancy monitoring, these biosensors offer significant advantages over traditional testing methods like ELISA. The combination of these advantages makes electrochemical biosensors a more efficient, faster, and more reliable diagnostic tool for clinical applications that require β-hCG detection. Despite these reported advantages, it is important to note that the analytical performance of electrochemical hCG/β-hCG biosensors remains highly dependent on the platform and is often demonstrated under optimized laboratory conditions. As a result, the translation of high sensitivity and a wide linear detection range into robust, reproducible, and clinically reliable devices continues to face several practical and engineering challenges, which will be discussed in the next section.

## 5 Research progress of existing sensors

The development of electrochemical biosensors for hCG detection has become an active area of research, with various approaches being explored. Recent advancements have demonstrated their versatility in detecting hCG in clinical samples. Several types of electrode modifications using conductive materials, different receptors and bioreceptors, as well as various electrochemical methods for detecting β-hCG have also been reported, as shown in Table 2. All the studies that have been conducted can be used for monitoring GTD or GTN diseases, but the detectable levels of hCG/β-hCG are limited to the range reported in that table.

**Table 2:** Electrochemical biosensor is designed and researched to detect beta hCG at levels above 500 mIU mL^−1^^a^

Electrode modification	Method	Limit detection (mIU mL^−1^)	Detection range (mIU mL^−1^)	Sample matrix	Reference
hCG-Fc-IgG complex	FIA	—	0–2000	Buffer	99
mAb_2_-(biotin-SA)-AP/hCG/mAb_1_-AuE	SWF	216	0–2000	Buffer	100
mAb_2_-(LC-SPDP)-AP/hCG/mAb_1_-AuE	SWF	240	0–4000	Buffer	100
mAb_2_-(LC-SPDP)-AP/hCG/mAb_1_-AuE	CA	175	0–5000	Buffer	101
Au/SiC-CS	DPV	0.042	0.1–5; 5–1000	Buffer & serum	102
Pd–Al alloy/HRP-Ab_2_/hCG/Ab1/GNPs/PB/GNps/GCE	CV	0.093	5–2000	Buffer & serum	103
Au/MWCNTs/GS	DPV	0.00286	0.005–500	Buffer & serum	104
SPEs/Ab_1_/GNPs-Ab_2_-ALP-IgG	DPV	0.36	1–100 000	Buffer & serum	105
LPCs-SnS_2_/AuNPs	CV	0.064	5–500	Buffer & serum	96
PANI/graphene-SPE	EIS	0.00286	0.01–500	Buffer & urin	106
AgNPs/Gr–IL–Chit	DPV	0.0066	0.0212–530	Buffer & serum	107
SPE/AuNP/peptideApt	EIS	1	5–1500	Buffer & serum	32
Au/Cys/peptide	EIS	0.0095	0.5–50 000	Buffer & serum	97
Graphene film on SiO_2_/Si substrate	FET	0.01	0.001–1000	Buffer & serum	108
Graphite/anti-rIgG/rIgG anti-β-hCG	CV	2.45	0–1000	Buffer & serum	109

a Abbreviations: Fc-IgG complex: ferrocenecarboxylic acid conjugated with anti-hCG immunoglobulin G antibody, mAb_2_: monoclonal second antigen-specific antibody, SA: streptavidin, AP/ALP: alkaline phosphatase, mAb_1_: monoclonal antigen-specific antibody, AuE: gold electrode, LC-SPDP: analyte-specific antibody on a self-assembled layer of recombinant protein G that was thiolated with succinimidyl-6-[3′-(2-pyridyldithio)-propionamido]hexanoate, SiC: silicon carbide, CS: chitosan, Pd–Al alloy foil: palladium-aluminium alloy foils, HRP: horseradish peroxidase, Ab_2_: secondary anti-hCG antibody, Ab_1_: anti-hCG, GNPs: gold nanoparticles, PB: Prussian blue, GCE: glassy carbon electrode, MWCNTs: multi-walled carbon nanotubes, GS: graphene nanosheets, SPEs: screen-printed electrodes, LPCs: lignin-based porous carbons, SnS_2_: tin disulfide, PANI: polyaniline, AgNPs: silver nanoparticles, Gr–IL–Chit: graphene–ionic liquid–chitosan, Apt: aptamer, Cys: cysteamine, SiO_2_: silicone dioxide, Si: silicone, anti-rIgG: anti imunoglobulin G form rabbit, rIgG anti-β-hCG: rabbit imunoglobulin G anti-β-hCG.

Laschi *et al.* (2023) developed an electrochemical biosensor for the detection of β-hCG in serum samples using graphite-based working electrodes were coated with anti-rIgG Fc. Anti-rIgG Fc acting as a blocking agent and enhancing the oriented immobilization of specific assay antibodies, rIgG anti-β-hCG. The detection mechanism involves a sandwich assay where the analyte interacts with two antibodies simultaneously, binding different epitopes of the molecule. The signal increases with the concentration of analyte present, and the extent of the specific reaction is measured by incubation with a solution containing a fixed concentration of the secondary rIgG anti-β-hCG-AP, reacting with a different β-hCG epitope. This results in a signal directly proportional to the antigen concentration, increasing at higher β-hCG concentrations. The calibration curve for β-hCG was reported in the range of 0–1000 mIU mL^−1^ for the electrochemical immunoassay at optimized conditions and limit of detection (LOD) for beta hCG detection was found to be 2.45 mIU mL^−1^.^109^

Koterwa *et al.* (2023) successfully developed an electrochemical biosensor by modifying a gold electrode with cysteamine and oligopeptide. Cysteamine was used as a linker between the gold electrode and the receptor, while the oligopeptide receptor was designed to specifically bind to hCG. The oligopeptide sequence utilized was Proline–Proline–Leucine–Arginine–Isoleucine–Asparagine–Arginine–Histidine–Isoleucine–Leucine–Threonine–Arginine. This biosensor demonstrated effective detection of hCG levels in human serum using Electrochemical Impedance Spectroscopy (EIS). The LOD was reported to be 0.0095 mIU mL^−1^, with a detection range spanning from 0.5 to 50 000 mIU mL^−1^.^97^

For hCG concentrations exceeding 50 000 mIU mL^−1^, a study by Cao *et al.* (2017) reported a highly sensitive electrochemical biosensor utilizing a printed-layer electrode functionalized with aldehyde groups to immobilize the capture antibody (Ab1). Additionally, a signal antibody (Ab2) was conjugated with gold nanoparticles (GNPs) to enhance conductivity and electrochemical signals. To further amplify the detection signal, alkaline phosphatase (ALP-IgG) was conjugated with a secondary antibody. These components were incorporated into a paper-based microfluidic system (µPADs), enabling efficient and rapid detection. The biosensor achieved a limit of detection (LOD) of 0.36 mIU mL^−1^, with a linear detection range from 1.0 mIU mL^−1^ to 100 000 mIU mL^−1^.^105^

Another significant innovation is the use of carboxylated and oxidized diamond nanoparticles in the development of immunosensors for β-hCG detection. Studies have shown that these diamond nanoparticles significantly enhance the sensitivity of the immunosensors, enabling more accurate detection even of trace amounts of β-hCG in biological samples.^110^ The unique properties of diamond nanoparticles, such as their high surface area and excellent conductivity, contribute to their ability to enhance electrochemical signals. This advancement is crucial in clinical settings where early detection of β-hCG is essential, such as in monitoring early-stage pregnancies or diagnosing gestational trophoblastic tumours.

In addition to nanomaterials, the development of label-free biosensors using graphene-based field-effect transistors (FETs) represents a breakthrough in biosensor technology. Graphene, known for its exceptional electrical conductivity and high surface-to-volume ratio, enables the creation of biosensors with outstanding sensitivity and specificity. Label-free sensors eliminate the need for additional labeling molecules, reducing complexity and cost while maintaining high levels of accuracy. These graphene-based FET biosensors have shown promising results in detecting β-hCG with superior sensitivity, making them valuable tools for early diagnosis in various medical applications, including reproductive health and oncology.^111^ These breakthroughs emphasize the potential of advanced materials in revolutionizing the future of biosensor technology.

Based on the research results that have been presented and the data summarized in Table 2, it can be concluded that electrochemical biosensors for the detection of hCG/β-hCG generally show very good analytical performance, particularly in terms of sensitivity and low detection limits. This performance is achieved thru various strategies of electrode and bioreceptor modification that effectively enhance electron transfer efficiency and biomolecule binding capacity. However, at medium to high quantification levels, most reported platforms still show a relatively limited linear dynamic range (LDR), generally only up to around 1000 mIU mL^−1^, as demonstrated in sandwich configuration-based studies. These limitations are likely caused by the saturation of the working electrode surface and signal limitations that arise due to the high bioreceptor density at increased antigen concentrations, as reported by Laschi *et al.* (2023).^109^

For comparison, electrodes modified using a self-assembled monolayer (SAM) approach based on thio-gold with oligopeptide linkers, combined with EIS detection methods, have been reported to detect hCG up to 50 000 mIU mL^−1^ with stable analytical performance. Although the use of small-sized synthetic receptors provides the advantage of reducing steric hindrance and delaying surface passivation, the impedance-based transduction mechanism remains susceptible to signal saturation at very high antigen loads, ultimately limiting the extension of the LDR (Koterwa *et al.*, 2023).^97^ On the other hand, the platform developed by Cao *et al.* (2017), which combines microfluidic paper with DPV measurement methods, demonstrates quantification capabilities up to 100 000 mIU mL^−1^.^105^ These findings highlight the crucial role of faradaic current-based signal amplification in enabling the quantification of hCG at high concentrations relevant to GTD. Overall, this comparison indicates that although many platforms excel in sensitivity, only biosensors that combine high binding capacity with transduction modes tolerant to signal amplification can bridge the gap between pregnancy level detection and pathological level monitoring in GTD.

## 6 Challenges and limitations of electrochemical biosensors

Although GTD arises in the context of pregnancy and is associated with hCG/β-hCG production, the concentration range, secretion dynamics, and molecular heterogeneity of hCG differ substantially between these conditions. GTD is characterized by heterogeneous hCG isoforms and variable concentration profiles that may overlap with physiological pregnancy levels, particularly in partial moles and certain trophoblastic tumours, limiting the discriminative value of pregnancy-oriented assays.^112–114^ Moreover, GTD is defined by abnormal genetic and histopathological features rather than pregnancy itself, and may occur in the absence of an ongoing pregnancy, necessitating quantitative hCG monitoring and comprehensive clinical evaluation for accurate diagnosis and risk stratification.^115–117^

At the analytical level, most of the electrochemical biosensors currently being developed for hCG detection are primarily optimized for concentration ranges relevant to early pregnancy. As a result, this platform has not yet been directly applicable for the quantification of hCG at very high concentrations (>10^5^ mIU mL^−1^), which are often associated with GTD, nor for certain tumour subtypes that produce relatively high levels of hCG/β-hCG and a broader spectrum of hCG isoforms. At high hCG levels, biosensing on electrodes has limitations due to the working electrode being prone to bioreceptor saturation, hook effect, and signal plateauing, while commonly used transduction modes (*e.g.*, EIS or DPV) lose responsiveness after the electrode interface is passivated.^118,119^

The development of electrochemical biosensors for β-hCG detection has made significant strides; however, several challenges and limitations persist that hinder their widespread clinical application. These challenges include issues related to linearity range, stability, reproducibility, interference from other biological molecules, and scalability for manufacturing.

Although electrochemical biosensors have demonstrated excellent performance in detecting hCG at relatively high concentrations, challenges remain in measuring extremely high levels. In cases of GTD, hCG concentrations can exceed 100 000 mIU mL^−1^, while in non-trophoblastic neoplasms, levels range between 1900 and 160 000 mIU mL^−1^.^73^ If biosensors are unable to detect hCG within these ranges, the early diagnosis and disease monitoring process may become suboptimal. Therefore, further technological advancements are needed to expand the detectable concentration range while maintaining accuracy and analytical robustness.

**Fig. 4:**
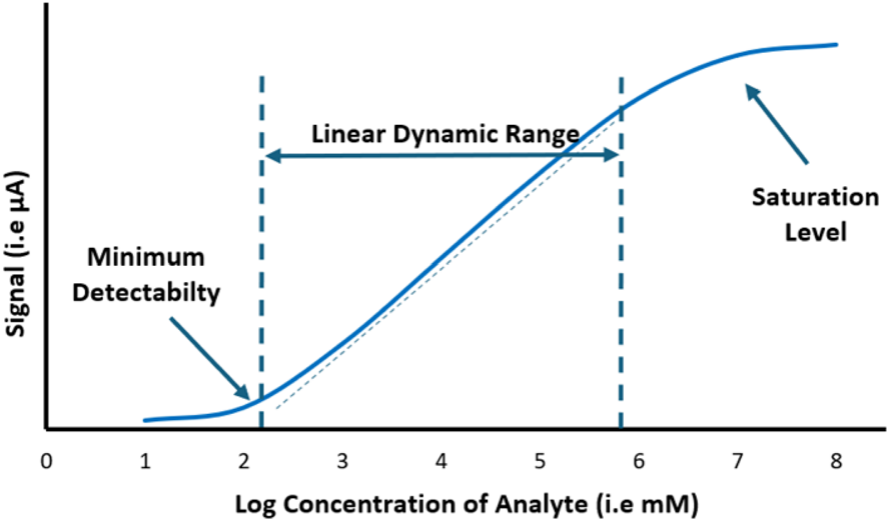
The demonstrated dynamic range in a biosensor standard curve, presenting the dose–response fitting curve.

One of the main analytical limitations in the electrochemical detection of hCG/β-hCG is sensor saturation at high antigen loads, which directly disrupts measurement accuracy. The linear range refers to the concentration range within which a biosensor can quantitatively measure hCG, from the limit of detection (LOD) to the point where the sensor becomes saturated, similar to the dynamic range (Fig. 4). While a wider dynamic range describes the overall signal window between the minimum and maximum detectable concentrations, proportionality is not guaranteed across this range, especially at very low or high concentrations. For this reason, the concept of LDR is more relevant for quantitative clinical diagnostics.

There is a fundamental trade-off between the linear range and biosensor sensitivity. Biosensors with very high sensitivity often exhibit a narrower detection range, as the signal saturates more rapidly. Conversely, biosensors with a wider linear range typically exhibit lower sensor sensitivity.^120^ To address these limitations, further innovations are required, including electrode material optimization, signal amplification enhancement, and transducer design aimed at delaying saturation and maintaining a proportional signal response at clinically relevant levels of hCG/β-hCG. Several studies have attempted to overcome these challenges by modifying biological recognition elements and electrochemical transducers in biosensors. The incorporation of nanomaterials and enzymatic amplification strategies has proven capable of increasing sensitivity while also extending, albeit still limited, the range of detectable hCG/β-hCG levels. However, additional challenges remain, particularly in ensuring long-term sensor stability and maintaining consistent performance when tested on clinical samples under diverse conditions.

In this context, sensor stability emerges as one of the main challenges for electrochemical biosensors. The performance of these sensors can degrade due to environmental factors such as temperature fluctuations, humidity, and the presence of interfering substances in biological samples.^121,122^ Specifically, the stability of electrodes modified with nanomaterials can vary significantly, ultimately leading to shifts in electrochemical response and reducing the analytical reliability of the device.^123^ This instability becomes increasingly critical in clinical applications that require repeated and long-term monitoring.

In addition to stability, reproducibility is also a crucial aspect in clinical diagnostics. Variations in fabrication process, such as differences in bioreceptor immobilization, can lead to inconsistent results.^121,124^ This lack of reproducibility can compromise the reliability of biosensors, especially in high-risk applications such as GTD diagnosis and therapy response monitoring, where clinical decisions heavily rely on the reliability of quantitative data.

The challenges of translation are further complicated by issues of scalability and manufacturability. Although advancements in materials science and nanotechnology have enabled the development of high-sensitivity laboratory biosensors, the fabrication process often involves complex and expensive materials or stepwise assembly techniques that are difficult to apply at mass production scale.^121,122^ At the manufacturing stage, strict quality control is required to ensure consistent performance across production batches, thereby adding complexity and cost to large-scale applications.^121,123,124^

In addition to technical and manufacturing issues, interference from biomolecules is also a major challenge when developing electrochemical biosensors for detecting hCG/β-hCG. Complex biological matrices, such as serum, plasma, urine, and saliva, contain various proteins, metabolites, and electroactive species that can generate background noise or cause nonspecific adsorption on the electrode surface, thereby reducing the accuracy of the measured signal.^125,126^ Additionally, cross-reactivity with hormones or biomolecules structurally similar to β-hCG poses a significant challenge for electrochemical biosensors.^127^

These interfering compounds are often present in complex biological matrices, which can lead to false-positive or false-negative results, ultimately reducing the accuracy of the biosensor.

Therefore, the creation of highly selective bioreceptors capable of distinguishing β-hCG from structurally similar molecules, combined with effective surface-blocking strategies and anti-fouling approaches, remains a key prerequisite for achieving reliable and accurate clinical diagnostics.^123,128^

With the advancement in electrode material engineering, signal amplification strategies, and biosensor technology, future platforms are expected to achieve reliable detection of hCG across a broader range, including concentrations exceeding 100 000 mIU mL^−1^, while ensuring long-term stability and high specificity. This advancement will provide significant benefits for GTD and GTN diagnosis and therapeutic monitoring, ultimately enhancing treatment effectiveness and patient prognosis. Therefore, further research in this field is crucial to overcome existing limitations and promote the wider application of electrochemical biosensors in medical diagnostic.

## 7 Strategies to improve electrochemical biosensor performance for hCG detection in GTD

Several strategies and approaches have been developed to enhance the analytical performance of electrochemical biosensors for the detection of hCG or β-hCG in GTD and GTN. Conventional electrochemical sensing platforms often face limitation such as insufficient sensitivity, limited linear dynamic range, inefficient charge transfer, and signal transduction at high analyte concentrations. To address this challenges, various optimization approaches have been explored, particularly in electrode material engineering, transducer and bioreceptor modification, and signal amplification strategies. These approaches play a crucial role in improving the sensitivity and reliability of hCG detection by enhancing interfacial charge transfer, improving biomolecule immobilization, maintaining proportional signal transduction, and delaying saturation. As the result, the biosensor performances can be significantly improved in term of sensitivity, signal stability, and linear dynamic range. Importantly, these improvements help minimize electrode saturation and reduce the risk of high-dose hook effects at clinically relevant hCG concentrations, thereby supporting the development of more robust and clinically applicable biosensing platform.

### 7.1 Electrode interface and material engineering for linear range extension

**Fig. 5:**
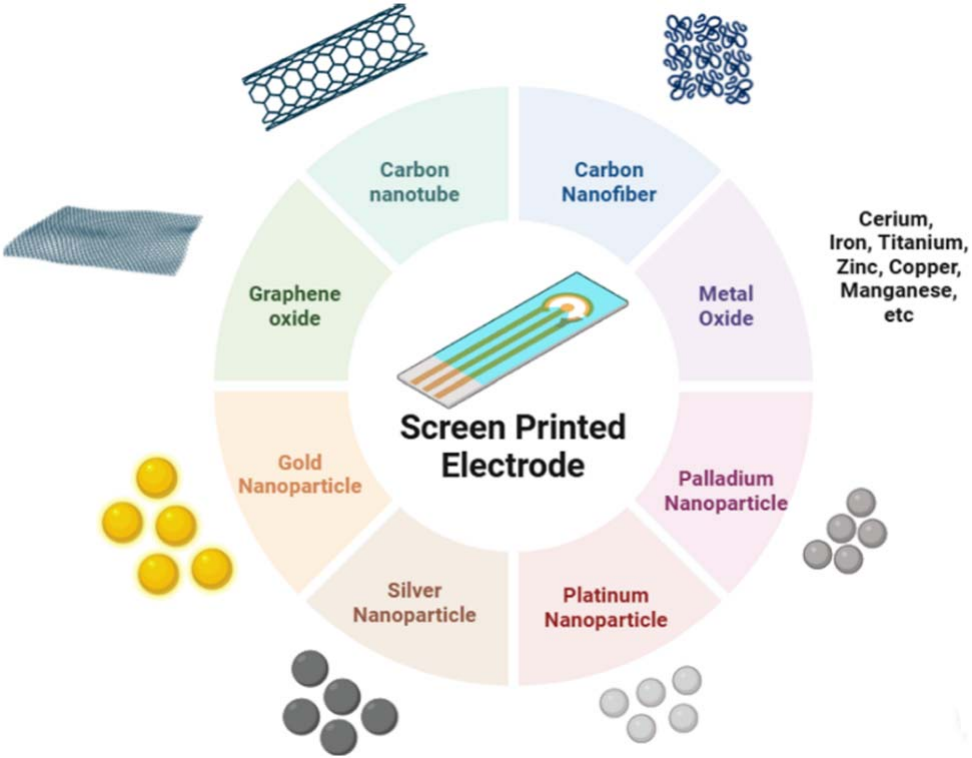
Illustration of screen-printed electrodes (SPE) modified with various nanomaterials to improve electrochemical performance.

One of the key factors governing the analytical performance of electrochemical biosensors is the electrode interface and materials engineering, which directly influences sensitivity, selectivity, and the attainable linear dynamic range.^129^ Among the various conductive materials investigated, nanomaterials have emerged as particularly promising candidates (Fig. 5), owing to their high surface area-to-volume ratio, superior chemical activity, and enhanced electrocatalytic properties compared to bulk-scale materials.^130^ The integration of conductive nanomaterials, such as carbon-based nanomaterials and metal nanoparticles, has been demonstrated to significantly enhance electron transfer kinetics while simultaneously lowering detection limit.^131,132^ These performance gains are primarily attributed to the enlarged effective surface area, the high density of electroactive sites, and improved charge-transfer efficiency at the electrode–electrolyte interface, which collectively enhance signal sensitivity and support the extension of the linear response range.

At elevated hCG concentrations, conventional electrochemical biosensors frequently suffer from signal saturation, manifested as response plateauing, and in certain immunoreaction-based systems this condition can further increase susceptibility to the high-dose hook effect. This behaviour is primarily attributed to limitations in active surface area, steric hindrance arising from high bioreceptor density, and mass-transport constraints at the electrode–electrolyte interface.^133,134^ In this context, high-surface-area nanostructured electrodes enable a more effective spatial distribution of binding sites and delay interfacial saturation, thereby preserving proportional signal responses even at elevated analyte concentrations. Accordingly, optimisation of nanomaterial synthesis routes and integration strategies has been increasingly recognised as an effective and validated approach to mitigating saturation effects and extending the linear dynamic range, without altering the underlying principles of biological recognition.^133,135,136^ Nevertheless, challenges related to material uniformity, long-term stability, and reproducibility remain critical barriers to the development of reliable biosensing platforms. Researchers have studied carbon-based nanomaterials like carbon nanotubes (CNTs) and graphene because they have high electrical conductivity, a large surface area, and mechanically flexible.^137,138^ These features help electrons move efficiently between biological recognition elements and the electrode surface, thereby making biosensors more sensitive and improving their electrochemical response. Carbon-based nanomaterials can also be combined with inorganic nanoparticles, conductive polymers, and biomolecules to create nanocomposites that are even more sensitive and selective. These hybrid systems have performed better in detecting biomolecules such as glucose, cholesterol, and cancer biomarkers.^139^ Three-dimensional (3D) carbon structures, including carbon nanowalls and diamond-based structures coated with metal oxide, such as CuO, have also been shown to enhance electrocatalytic activity and stability by accelerating mass and charge transport.^140^

In addition to carbon-based nanomaterials, metal nanoparticles such as gold (Au), silver (Ag), platinum (Pt), and palladium (Pd) have been extensively investigated as conductive modifiers for electrochemical biosensors. These nanoparticles possess a high specific surface area, strong electrocatalytic activity, and efficient electron transfer properties, which collectively enhance analytical sensitivity and lower detection limits.^138^ Among these, gold nanoparticles (AuNPs) are the most frequently utilized. Electrodes modified with AuNPs have demonstrated up to 70% greater sensitivity for hydrogen peroxide detection compared to unmodified electrodes.^141–143^

Silver nanoparticles (AgNPs) also exhibit significant potential, increasing electron transfer efficiency by approximately 40% or more.^144,145^ This is consistent with that reported by Sharma *et al.* (2024) in their study on aptamer biosensors for the Chikungunya virus.^144^ Platinum nanoparticles (PtNPs) and palladium nanoparticles (PdNPs) (PdNPs) further enhance current response and sensitivity, with PdNPs reported to extend the linear detection range up to 0.05–50 mM.^146–149^ Uniform distribution of metal nanoparticles on substrates generates additional electroactive sites, thereby delaying signal saturation and providing more attachment points for bioreceptors. This approach reduces crowding and prevents premature signal saturation.^133,135,150,151^

Furthermore, metal oxide–carbon composites have been widely recognized as promising electrode materials for expanding the linear detection range in electrochemical biosensors. This material combines the high electrocatalytic activity of metal oxide nanostructures with the superior electrical conductivity and large surface area of carbon nanomaterials.^152–154^ The synergy between metal oxides or mixed-metal derivatives (oxides/sulfides/selenides) and functionalized carbon not only enhances electron-transfer kinetics but also provides a more effective interface for bioreceptor immobilization, thereby supporting more proportional signal transduction across a wide range of analyte concentrations. Various studies show that TiO_2_, ZnO, SnO_2_, WO_3_, and Fe_2_O_3_-based composites combined with conductive polymers or graphene exhibit broader current responses and a wider linear range than single-component electrodes.^155–158^ In the context of immunosensors, increased active surface area and mass transport efficiency can delay the decrease in signal response at very high analyte concentrations, a phenomenon often associated with the high-dose hook effect.

The effectiveness of this approach is further enhanced by the use of three-dimensional (3D) electrodes, which enhance both analyte diffusion and electron-transfer efficiency. A variety of 3D materials, including those based on metals, metal oxides, carbon, and silicon, have demonstrated superior electrochemical performance due to their large specific surface area, porosity, and interconnected three-dimensional conductive pathways.^133,134,159,160^ These porous architectures accelerate reaction kinetics and minimize mass-transport limitations that can lead to signal saturation at elevated analyte concentrations. Furthermore, the multiple conductive pathways present in structures such as 3D monolithic graphene foam facilitate efficient signal transduction from binding sites distributed throughout the electrode volume, even under high analyte loads. The alignment of nanostructures, such as vertically oriented carbon nanotubes (CNTs), increases the accessibility of the active surface and supports non-planar radial diffusion, which helps maintain signal linearity. Therefore, the combined engineering of materials and electrode architecture not only improves biosensor sensitivity but also extends the linear detection range of hCG/β-hCG to clinically relevant high concentrations.

Additionally, integration strategies such as direct deposition, electrochemical deposition, and layer-by-layer assembly enable precise control over electrode morphology.^130,161,162^ The ability to regulate the density and spatial orientation of bioreceptors through these methods is a key factor in designing optimal transducers and biological recognition elements. Such control is crucial for balancing sensitivity at low concentrations with response linearity at high analytical levels. Optimizing electrode materials, surface chemistry, and nanostructural architecture enables electrochemical biosensors to accommodate a wide dynamic range of hCG/β-hCG concentrations across various clinical scenarios. This material and structural foundation is an essential prerequisite for further system-level optimization, particularly in selecting transduction modes and biological recognition elements.

### 7.2 Transducer and bioreceptor selection for specific hCG recognition

Beyond electrode material engineering, the strategic selection of transduction methods and bioreceptors is essential to improving the analytical performance of electrochemical biosensors for detecting hCG/β-hCG in GTD. These components determine how biorecognition events at the electrode interface are translated into measurable electrical signals, directly influencing sensitivity, dynamic linear range, and vulnerability to signal saturation.

Common electrochemical transduction modes for β-hCG detection include amperometric, voltammetric, and impedimetric techniques, each characterized by distinct signal generation mechanisms. Amperometric and voltammetric methods utilize faradaic current responses and exhibit greater tolerance to high analyte concentrations, making them suitable for therapy monitoring and disease progression assessment. In contrast, impedance-based techniques enable label-free detection by tracking changes in interfacial resistance and capacitance, offering high sensitivity at low β-hCG concentrations but often exhibit nonlinear responses at elevated antigen levels due to surface passivation and increased charge-transfer resistance. Xu *et al.* (2023) and Deniz *et al.* (2025) report that tailoring the transduction mode to the specific matrix and clinical context, such as serum or urine, significantly enhances analytical reliability and operational range.^89,163^

The choice of bioreceptors is critical for determining the specificity and durability of biosensors across a range of analyte concentrations. Antibody-based recognition layers are the most established platform because of their high affinity and clinical validation. Nevertheless, the relatively large size of antibody molecules can result in steric hindrance, rapid receptor saturation, and signal plateauing at elevated antigen concentrations, particularly in sandwich-type immunosensors. These limitations are demonstrated in electrochemical immunosensor studies by Contreras Jiménez *et al.* (2015) and Laschi (2023), which associate the restricted linear dynamic range with the interface density and the limited charge-transfer capacity.^109,164^

Aptamer-based biosensors represent a mechanistically advantageous alternative because of their reduced size, conformational flexibility, and facile chemical modification. These characteristics facilitate a more uniform spatial distribution on electrode surfaces and enhance accessibility to binding sites. According to Dąbrowski *et al.* (2019), aptamers also exhibit greater chemical stability than antibodies, thereby supporting reproducible signal transduction across diverse analytical conditions and minimizing steric effects at elevated β-hCG concentrations.^98^

Molecularly imprinted polymers (MIPs) expand the design space by incorporating synthetic recognition elements with distributed binding cavities. In contrast to discrete bioreceptor binding, MIP-based systems partially decouple signal generation from receptor occupancy, thereby reducing vulnerability to high-dose hook effects and enabling reliable quantification under antigen-excess conditions. Laschi *et al.* (2023) highlight that this receptor architecture provides a promising approach for achieving a broader linear dynamic range.^109^

Careful regulation of binding kinetics and bioreceptor density is essential for suppressing signal saturation. The spatial arrangement of bioreceptors on structured electrode surfaces, such as micropillar array architectures, further mitigates steric effects and enhances effective binding events.^165,166^ Collectively, optimizing both transduction modes and bioreceptor architecture establishes a mechanism-based framework to address the underlying causes of signal saturation in electrochemical biosensors. This approach also provides a critical foundation for the development of advanced signal amplification strategies.

### 7.3 Signal amplification approaches

Signal amplification is one way to overcome several analytical challenges inherent in biosensor systems, such as receptor saturation, nonlinear signal behavior, and high-dose hook effects, which can reduce measurement accuracy and reliability. In electrochemical biosensors, amplification is particularly effective because it shifts signal generation away from direct analyte–electrode interactions, allowing for a much wider linear dynamic range. This capability is particularly important for hCG/β-hCG analysis in GTD because clinically relevant antigen concentrations are often high and require precise quantification. Under these conditions, analyte levels often exceed the linear response window of conventional immunoassays and many previously reported electrochemical biosensor platforms.

Enzymatic signal amplification is a well-established approach that utilizes catalytic reactions mediated by enzymes such as endonuclease and alkaline phosphatase (ALP) to generate multiple signal molecules from a single biorecognition event. This catalytic turnover enhances detection sensitivity and reduces receptor saturation and nonlinear responses by shifting signal generation downstream of the binding event and confining it to the surface. Recent studies indicate that enzymatic amplification substantially improves biosensor performance under challenging analytical conditions, especially when quantifying low-abundance targets within complex backgrounds.^167^

In addition to enzymatic approaches, nanoparticle-based signal amplification leverages the distinct electronic and electrochemical properties of nanomaterials, including gold nanoparticles and quantum dots. These nanostructures increase the loading capacity for redox-active or enzymatic labels, thereby strengthening the detected signal and enabling a more linear response over a broader range of analyte concentrations. Distributing signal generation across multiple amplification centers, rather than relying exclusively on receptor occupancy, allows nanoparticle-assisted strategies to reduce susceptibility to high-dose hook effects.^167^

Advanced architectures employ multi-stage and hybrid amplification strategies to further increase sensitivity and dynamic range. The staged approach, exemplified by the metasurface plasmon resonance scheme (MSA-metasPR), integrates sequential amplification steps to maximize signal output, thereby reducing receptor saturation and minimizing nonlinear behavior at elevated analyte concentrations.^168^ Hybrid systems combine enzymatic transduction mechanisms, nanoparticle-based methods, and label-free techniques, leveraging the strengths of each to maintain signal proportionality across a broad concentration range and effectively mitigate the hook effect.

**Fig. 6:**
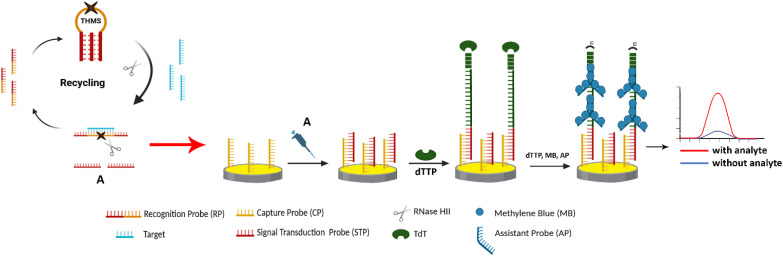
Schematic representation of enzyme-assisted electrochemical signal amplification, which employs coupled enzymatic reactions and redox-labelled DNA architectures. This amplification cascade generates signals independently of surface-limited bioreceptor binding, thereby reducing signal saturation and expanding the linear dynamic range at elevated analyte concentrations (adapted from reference Wang *et al.* 2018 with permission from Elsevier, copyright 2018).^169^

The combination of enzymatic reactions with electrochemical–chemical or electrochemical–chemical–chemical redox cycles has emerged as a highly effective strategy in electrochemical biosensors. This approach enables highly sensitive detection by simply adding one or two additional chemical reagents to the solution, without the need for extra enzymes or electrode modifications. The primary advantage of this method lies in its simplicity, as it enhances detection sensitivity without requiring complex system modifications, show in Fig. 6.^169,170^

In an enzymatic redox cycle system, the presence of the target analyte triggers a series of enzymatic reactions that produce or consume redox-active species, which can be detected by an electrochemical process. These mechanisms have been extensively validated in nucleic acid-based biosensing platforms. For instance, a dual-enzyme-assisted amplification system utilizing RNase HII and terminal deoxynucleotidyl transferase (TdT) achieves ultrahigh sensitivity by enabling target recycling and enzymatic signal amplification. In a representative approach, a triple-helix molecular switch (THMS), consisting of a signal transduction probe (STP) and a recognition probe, acts as a molecular recognition and signal transduction unit. After recognition of target ctDNA, RNase HII facilitates the disassembly of the THMS, releasing several STPs for target recycling amplification (A). Second, the released STPs bind to the capture probe on the electrode surface, triggering TdT-mediated DNA polymerization to generate long single-stranded DNA (ssDNA) “stems,” as TdT catalyzes the addition of dNTPs at the 3′-OH ends. Third, a specially designed assistant probe (AP) initiates a second TdT-mediated extension, forming a branched DNA structure decorated with redox-active methylene blue (MB). The generated electrochemical signal is directly proportional to the amount of MB-labeled DNA, ensuring high sensitivity and effective target detection through enzymatic amplification and electrochemical readout.^171^

Adapting enzymatic amplification schemes from nucleic acid detection to protein biomarkers, such as β-hCG, presents distinct mechanistic challenges. Unlike nucleic acids, proteins lack inherent sequence complementarity and therefore require indirect signaling *via* antibodies, aptamers, or affinity tag-based recognition interfaces. This additional layer of recognition can introduce steric hindrance, reduce amplification efficiency, and increase susceptibility to nonspecific background signals, especially at high antigen concentrations. This results in multi-stage enzymatic cascades that can amplify both target-derived and nonspecific signals if not tightly controlled.

Despite these challenges, enzyme-assisted amplification strategies with conceptual similarities have been successfully implemented in protein biosensing. The predominant method for signal amplification in protein biosensors involves enzyme- or nanozyme-based catalytic amplification, in which antibodies recognize the target protein, and the enzymatic or nanozyme label produces multiple signal products from a single binding event. This approach is widely utilized in immunoassays and contemporary biosensor platforms. For instance, the MOF-Au nanozyme, in conjunction with antibodies and DNA aptamers, facilitates the detection of tau and BACE1 proteins at femtogram per milliliter levels through radical generation and electrochemical dye catalysis.^172,173^ Enrichment of horseradish peroxidase (HRP) labels using phage and anti-phage systems enables substantial dual-signal amplification for SARS-CoV-2 spike protein detection.^174^ Similarly, in impedance-based electrochemical systems, the combination of Cu-MOF peroxidase nanozyme and glucose oxidase (GOx) enzyme initiates radical polymerization and markedly increases impedance for the detection of the biomarker CA15-3.^175^

Beyond catalytic schemes, luminescence-based platforms, including electrochemiluminescence (ECL) and time-resolved fluorescence (TRF), offer alternative, highly effective amplification strategies for protein analysis. Europium-based metal–organic frameworks (Eu-MOFs), which exhibit intrinsic luminescent properties, have been used as ECL probes in combination with antibody probes to achieve ultrasensitive antigen detection.^176^ In the context of time-resolved fluorescence immunoassays (TRFIA), two-stage amplification strategies that combine tyramide signal amplification (TSA) with the release of multiple europium ions from nanoparticle carriers have enabled highly sensitive detection of anti-Müllerian hormone (AMH) with markedly improved signal-to-background ratios.^177^

An increasingly prominent strategy involves coupling protein recognition events to nucleic acid amplification processes. In such systems, antigen binding by antibodies or aptamers triggers downstream DNA or RNA amplification reactions, including T7 transcription, hybridisation chain reaction (HCR), or strand displacement amplification (SDA), such that the measurable signal originates from amplified nucleic acids rather than directly from the protein target.^177–179^ Representative examples include protein-responsive aptamers that activate T7 transcription to generate large quantities of fluorescent RNA for multiplex protein detection in serum, as well as “turn-on” RNA aptamer systems for sensing anti-digoxin antibodies.^180,181^ In strip-based diagnostics, universal amplification concepts have also been realised, exemplified by “all-in-one” lateral flow immunoassays (LFIA) that exploit biotin–streptavidin interactions combined with *in situ* metal growth to achieve triple signal amplification for *Treponema pallidum* antibody detection.^182^

From a mechanistic perspective, enzymatic amplification and redox cycling are particularly advantageous for β-hCG detection, as they decouple signal generation from surface-bound receptor-binding events. By amplifying downstream electrochemical processes rather than relying exclusively on receptor occupancy, such approaches effectively extend the linear dynamic range and suppress signal saturation at high analyte concentrations. As schematically illustrated in Fig. 6, enzymatic amplification coupled with redox cycling redistributes signal generation into the solution phase, thereby alleviating surface saturation effects while maintaining proportional signal evolution under antigen-excess conditions.

Overall, signal amplification provides a powerful and adaptable framework for improving the sensitivity, linearity, and robustness of electrochemical biosensors. Although some of the approaches discussed in this section have been validated primarily for nucleic acid detection, their underlying mechanistic principles are directly relevant to β-hCG detection. Continued efforts to translate and optimize these strategies for protein biomarkers are expected to play a crucial role in overcoming signal saturation and dynamic range limitations in electrochemical biosensors for GTD diagnosis and therapy monitoring.

## 8 Future prospects of electrochemical biosensors for detection of β-hCG eta in GTD

Electrochemical biosensors for β-hCG detection hold promising potential due to the increasing demand for rapid, accurate, and user-friendly diagnostic methods. This technology continues to evolve, particularly in improving its sensitivity and specificity. Beyond pregnancy detection, β-hCG-based biosensors also play a crucial role in diagnosing specific diseases, such as GTD. Recent technological innovations in electrochemical biosensors have focused on enhancing the sensitivity and specificity of β-hCG detection, which is critical for improving diagnostic accuracy. One of the major advances in this field is the incorporation of nanomaterials, such as gold nanoparticles and carbon-based materials. These nanomaterials play a crucial role in amplifying electrochemical signals, thereby increasing the detection capability of the biosensors. Additionally, they enhance the immobilization of bioreceptors, which are responsible for capturing and recognizing the target β-hCG molecules. Recent advancements in bioreceptor immobilization techniques have demonstrated a notable enhancement in stability and precision. This development has led to significant improvements in biosensor performance, particularly in the detection of low target concentrations. This advancement renders biosensors highly effective for medical diagnostics.^183^

Another innovation driving the advancement of electrochemical biosensors is microfluidic technology, which enables high-efficiency sample analysis in small volumes while significantly reducing detection time. The integration of microfluidics into electrochemical biosensors has significantly transformed the field of diagnostics by enabling the development of miniaturized and automated testing platforms. Microfluidic systems allow for precise manipulation and control of fluid movement, which is essential for accurate sample handling and β-hCG detection, particularly in clinical environments. This precision is crucial in clinical applications where small sample volumes are often required, and maintaining accuracy is of utmost importance. The ability to control sample flow and reaction conditions within a microfluidic device enhances the reliability of biosensor readings, making them highly suitable for β-hCG detection in various clinical settings.^184,185^

Moreover, microfluidic devices can be designed to perform multiple diagnostic tests simultaneously, streamlining the diagnostic process and providing rapid results. This capability is particularly advantageous in cases where multiple biomarkers need to be analyzed concurrently, such as in reproductive health or cancer diagnostics. Customized paper-based microfluidic platforms, for instance, have been developed specifically for colorimetric immunosensing of β-hCG, offering a simple yet effective solution for use in low-resource or remote settings. These platforms not only reduce the cost of diagnostics but also improve accessibility, allowing healthcare providers to deliver essential testing in underserved regions where traditional diagnostic equipment may not be available.^184^

Additionally, the integration of POC devices allows these biosensors to be used directly in healthcare facilities, expediting the diagnostic process and enabling faster medical decision-making. The evolution of POC devices, which often incorporate microfluidic technology, has further revolutionized clinical diagnostics by enabling bedside testing. These portable, user-friendly devices have significantly reduced the time required for making critical diagnosis and treatment decisions. In emergency settings, where timely and accurate information is essential for patient care, POC biosensors can deliver immediate results, leading to faster clinical decisions and improving patient outcomes. This rapid on-site testing capability is particularly beneficial in scenarios such as pregnancy monitoring, where early and reliable detection of β-hCG levels can have a significant impact on patient management and treatment plans.^186,187^

Another significant advancement in the development of electrochemical biosensors is their potential for multiplex detection. Multiplexing refers to the ability of a biosensor to detect multiple biomarkers simultaneously, which provides a more comprehensive diagnostic profile in a single test. This capability is particularly valuable in medical diagnostics, as many conditions require the assessment of more than one biomarker for an accurate diagnosis. For example, in the case of ectopic pregnancy, measuring only β-hCG may not be sufficient for a definitive diagnosis. However, by simultaneously detecting other relevant biomarkers, clinicians can gain a broader understanding of the patient's condition, thereby improving diagnostic accuracy and ensuring more informed medical decisions.^188^ The multiplexing ability of electrochemical biosensors thus enhances their utility in complex diagnostic scenarios.

Multiplexed biosensors are particularly useful in situations that require monitoring changes in multiple biomarkers over time. For example, in the diagnosis and management of cardiovascular disease, levels of biomarkers such as troponin, B-type natriuretic peptide (BNP), and C-reactive protein (CRP) can fluctuate significantly. This variability makes tracking these markers simultaneously essential to gain a complete understanding of a patient's health. Similarly, in the diagnosis and management of ectopic pregnancy, multiple biomarkers such as β-hCG, progesterone, and other hormone levels can fluctuate, requiring simultaneous monitoring to gain a complete clinical picture. Using multiplexed techniques, healthcare providers can gain a more comprehensive picture of a patient's condition, allowing for earlier and more accurate intervention. Additionally, assessing multiple biomarkers in a single test reduces the need for multiple blood draws, reducing patient discomfort, saving time, and lowering the overall cost of the diagnostic process.^189–191^

Moreover, continuous monitoring of β-hCG levels has now become feasible with the advancements in electrochemical biosensors, enabling real-time tracking of hormonal fluctuations during pregnancy or in the context of specific cancers. This capability allows for ongoing observation of β-hCG levels without the need for repeated invasive testing, significantly enhancing patient comfort and care. Continuous monitoring can provide critical insights into the progression of a pregnancy or the development of cancer, allowing clinicians to intervene promptly when abnormal changes are detected. This real-time data can lead to personalized treatment strategies, where therapy can be adjusted based on the patient's unique hormonal profile and biomarker dynamics. Additionally, early detection of complications through continuous monitoring can improve patient outcomes by enabling timely interventions that mitigate risks associated with delayed diagnosis or treatment.^66,189^

In the future, electrochemical biosensors for β-hCG are expected to advance further with improved electrode materials, more efficient bioreceptor immobilization methods, and integration with artificial intelligence (AI) for more accurate data analysis. With these innovations, this technology has the potential to become a more sophisticated, efficient, and widely accessible diagnostic solution.

## 9 Conclusions

Accurate quantification of β-hCG remains fundamental for diagnosis, therapeutic monitoring, and recurrence surveillance of GTD and GTN. While conventional immunoassays such ELISA and chemiluminescent assays remain the clinical standard, their reliance on centralized laboratories and time-consuming workflows limits their use for rapid clinical decision-making. Electrochemical biosensors have emerged as promising alternatives due to their rapid response, high sensitivity and potential for miniaturization into point-of-care diagnostic platforms. Recent advances in electrode materials, transducer engineering, and signal amplification strategies have significantly improved analytical performance. Nevertheless, challenges related to dynamic range, signal saturation at high hCG concentration, and long-term stability remain to be addressed. Future efforts should focus on clinically oriented sensor design, integration with portable diagnosis system, and validation in real clinical setting to enable reliable, real-time monitoring and ultimately support more accessible and personalized management of GTD and GTN.

## Author contributions

This manuscript was written through contributions from all authors. Conceptualization, writing of the original draft, and writing – review and editing, Y. N., and H. I. F.; writing – review and editing, W. W., and A. K.; supervision, G. N. A. W., Y. M. H., K. I. M., Y. W. H., T. S., and M. Y.; funding, M. Y. All authors have read and approved the published version of the manuscript. All authors have given their approval for the final version of the manuscript.

## Conflicts of interest

There are no conflicts to declare.

## Data Availability

No primary research results, software or code have been included and no new data were generated or analysed as part of this review.
